# Anti-Aging Effects of Calorie Restriction (CR) and CR Mimetics Based on the Senoinflammation Concept

**DOI:** 10.3390/nu12020422

**Published:** 2020-02-06

**Authors:** Dae Hyun Kim, EunJin Bang, Hee Jin Jung, Sang Gyun Noh, Byung Pal Yu, Yeon Ja Choi, Hae Young Chung

**Affiliations:** 1 Department of Pharmacy, College of Pharmacy, Pusan National University, 2, Busandaehak-ro 63 beon-gil, Geumjeong-gu, Busan 46241, Korea; bioimmune@hanmail.net (D.H.K.); eunjin2285@gmail.com (E.B.); king2046@hanmail.net (H.J.J.); rskrsk92@naver.com (S.G.N.); 2 Department of Physiology, The University of Texas Health Science Center at San Antonio, San Antonio, TX 78229, USA; 3 Department of Biopharmaceutical Engineering, Division of Chemistry and Biotechnology, Dongguk University, Gyeongju 38066, Korea

**Keywords:** aging, calorie restriction, senescence-associated secretory phenotype, senoinflammation, mimetics

## Abstract

Chronic inflammation, a pervasive feature of the aging process, is defined by a continuous, multifarious, low-grade inflammatory response. It is a sustained and systemic phenomenon that aggravates aging and can lead to age-related chronic diseases. In recent years, our understanding of age-related chronic inflammation has advanced through a large number of investigations on aging and calorie restriction (CR). A broader view of age-related inflammation is the concept of senoinflammation, which has an outlook beyond the traditional view, as proposed in our previous work. In this review, we discuss the effects of CR on multiple phases of proinflammatory networks and inflammatory signaling pathways to elucidate the basic mechanism underlying aging. Based on studies on senoinflammation and CR, we recognized that senescence-associated secretory phenotype (SASP), which mainly comprises cytokines and chemokines, was significantly increased during aging, whereas it was suppressed during CR. Further, we recognized that cellular metabolic pathways were also dysregulated in aging; however, CR mimetics reversed these effects. These results further support and enhance our understanding of the novel concept of senoinflammation, which is related to the metabolic changes that occur in the aging process. Furthermore, a thorough elucidation of the effect of CR on senoinflammation will reveal key insights and allow possible interventions in aging mechanisms, thus contributing to the development of new therapies focused on improving health and longevity.

## 1. Introduction

The aging process can be defined as progressive, physiological, functional deterioration throughout the lifetime of an individual by different convoluted interactions among genes and non-genetic environmental factors that eventually result in disruption of homeostasis and increased susceptibility to disease or death. The basic mechanism of the aging process is a sustained, long-term inflammatory state that is further aggravated by elevated oxidative stress due to enhanced reactive oxygen species (ROS), lipid peroxidation, and protein oxidative modifications [1]. To understand the phenomenon of aging and its significance, several concepts and terminologies have been proposed including inflammaging, molecular inflammation, micro-inflammation, pan-inflammation, and gero-inflammation. These concepts and terminologies are apposite for describing the increased chronic inflammatory events and mediators during aging [2,3,4,5,6].

Generally, cells are continually exposed to and damaged by exogenous and endogenous stress inducers. The cell cycle of damaged cells, which cannot be recovered from cell death, is permanently arrested and the proliferative activity of these cells becomes extinct, which is defined as cellular senescence. This cellular response largely contributes to an organism’s aging. Senescent cells have been shown to release multiple inflammatory cytokines and chemokines, which is defined as senescence-associated secretory phenotype (SASP) [7]. An increase in cellular dysregulation due to the release of proinflammatory molecules such as TNF, IL-1β, IL-6, MCP-1, MIP-1α, RANTES, and IL-18 [8,9] induces age-related chronic inflammation, leading to aging and its associated diseases.

In order to understand age-related chronic inflammatory progress from a multilayered point of view, we previously proposed a novel concept of senoinflammation, which includes an expanded systemic view of chronic inflammation during the aging process.

CR is a well-known gold standard for many aging intervention strategies. A number of age-related biological changes and pathologic abnormalities can be delayed or suppressed by CR regardless of gender and species (mammalian or non-mammalian) [10]. CR has been shown to suppress oxidative damage-induced alterations and age-related diseases and to extend lifespan [11]. In this review, we focus on the diverse protective effects of CR against aging from a senoinflammatory perspective. In addition, the beneficial effects of CR mimetics and other types of dietary restrictions on anti-aging are covered.

## 2. Age-related Inflammation and Senoinflammation

**Figure 1:**
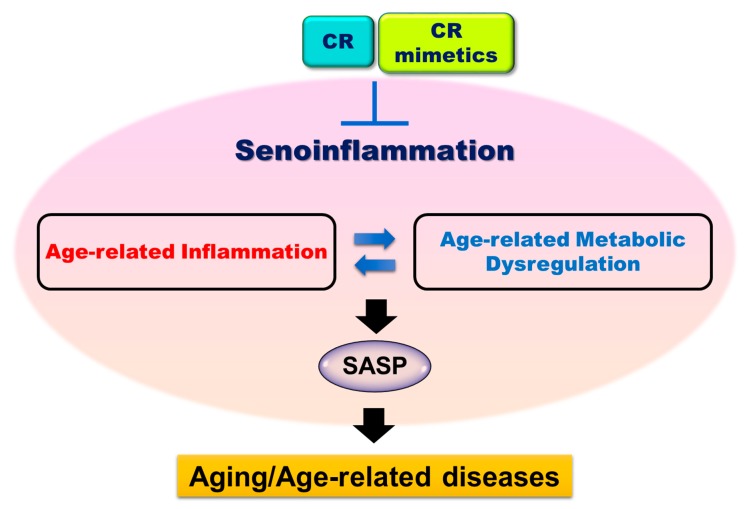
Possible mechanism of senoinflammation in aging. During aging, the continuous age-related inflammatory responses that cause metabolic dysregulation to form a vicious cycle. Age-related inflammation and metabolic dysregulation result in the induction and activation of many pro-inflammatory genes, metabolic and signaling pathways, and SASP. This vicious cycle of age-related chronic inflammation and metabolic dysregulation underlies the senoinflammation phenomenon, which occurs because of the interaction between senescent cells, immune cells, adipocytes, hepatocytes, and myocytes. SASP, senescence associated secretory phenotype; CR, calorie restriction.

Senescent cells produce proinflammatory senescence-associated (SA) secretome, which is referred to as the SASP. Macrophages are recruited in the secretome by chemotactic factors to clear senescent cells [12]. However, senescent macrophages with M2 polarized phenotype secrete proinflammatory cytokines and exhibit impaired phagocytosis and chemotaxis, and a downregulated rate of cellular proliferation [13,14,15]. It has been proposed that deficiency in the ability of aged macrophages to clear senescent cells leads to increased inflammatory response and results in chronic inflammation as SASP plays a role in the initiation of tissue inflammation [16]. Based on previous observations and evidence of the aging process at molecular and cellular levels, we coined the term “senescent inflammation (senoinflammation)” with a new framework (Figure 1) in our recent review to provide an expanded, broader view of age-related chronic inflammation and metabolic dysfunction [17].

Aging in hepatocytes is associated with various markers of cellular senescence, such as increased expression of heterochromatin protein 1β, and increased activity of senescence-associated-β-galactosidase, p21 and p16 [18]. p53 expression is an important marker of cellular senescence and DNA damage in the normal liver [19], and its regulation depends on the nutrient-sensing pathways of non-alcoholic fatty liver disease (NAFLD) [20]. Senescent hepatocytes exhibit increased lipid droplet accumulation and ROS production [21].

In understanding age-related inflammation at the molecular level, an abundance of data in our and other previous work strongly suggested that NF-κB is a key player involved in the initiation and exacerbation of tissue inflammation in the aging process and cancer [22,23,24]. Chronic transactivation of NF-κB has been observed in multiple tissues in various experimental models of aging and human fibroblasts and human CD4+ T cells obtained from aged individuals [25,26]. NF-κB induces proinflammatory mediators, chemokines, and adhesion molecules [27] and interacts with other transcriptional factors that are involved in the initiation and deterioration of chronic inflammatory response including signal transducers, the activator of transcription 3 (STAT3) and p53 [22]. The transcriptional activity of NF-κB occurs concurrently with crosstalk among upstream signaling components such as glycogen synthase kinase 3 (GSK3)-β, mitogen-activated protein kinase (MAPK), mammalian target of rapamycin (mTOR), and protein kinase B (PKB) [23,28].

The upregulation of systemic inflammation is associated with aging and age-related chronic diseases [29,30]. As mentioned, age-related systemic inflammation and senoinflammation are functionally distinct from acute inflammatory responses due to sustained high levels of pro-inflammatory mediators. In fact, epidemiological and experimental results suggest that persistent low-grade, chronic inflammation exists in aged animals [31,32]. A recent longitudinal, semi-supercentenarian study in Japan has demonstrated that inflammation, and not telomere length, strongly predicts successful aging at extremely old age [33]. This study concluded that chronic and systemic inflammation has a significant effect on mortality and had a correlation with the decline in cognitive function in the centenarians, thus showing that chronic inflammation is a critical risk factor in the aging process [33]. An increase in systemic inflammation is related to many aging phenotypes. For example, abruptly increased inflammation is generally associated with tissue dysfunction, metabolic syndrome, immune dysfunction, and neuronal complications [34].

CR animals live fairly longer with the right amount of nutrients, and most of the typical age-related chronic diseases are prevented or delayed in them. For example, the incidence of cancer, the main cause of death in rodents, is significantly reduced in CR animals. Similarly, reduced incidence or slower disease progression has been reported for cardiomyopathy, diabetes, chronic lung diseases, autoimmune diseases, and neurodegenerative diseases [35,36]. Preclinical and preliminary clinical studies have shown that CR or fasting can effectively prevent the development of malignant tumors through a variety of cellular responses and can improve the efficacy of therapeutics [37]. CR reverses most symptoms of immunosenescence, including decreased naïve T cell and increased memory T cell population [38], reduced T cell proliferative response to mitogens or antigens, reduced IL-2 production and NK activity [38,39,40,41,42], age-related increase in serum levels of TNFα and IL-6 [43], and autoimmune diseases [44,45]. More recently, CR was shown to delay immunosenescence in animals; however, this effect needs to be confirmed in humans.

Therefore, the concept of senoinflammation shows the orchestral performance of activated pro-inflammatory cytokines and transcription factors at a molecular level, immune cell senescence and SASP at a cellular level, and systemic inflammation and metabolic disorders at a systemic level during the aging process.

## 3. Calorie Restriction

In an initial study on CR by McCay et al. [46], the growth retardation hypothesis was investigated in a rat model by reducing food intake or CR, which slowed down the growth rate and prolonged lifespan. Various additional studies on CR have been conducted in diverse species ranging from yeast, fish, drosophila, hamsters, dogs, and non-human primates to humans [47]. Experts in the field of aging have accepted CR as an anti-aging experimental concept, which serves as an established aging intervention. As CR is a non-genetic, nutritional means to delay the aging process, it was used to identify underlying signaling mechanisms of aging, resulting in its considerable importance. Understanding elemental mechanisms underlying the effect of CR is critical as they may aid in identifying novel therapeutic molecular targets for age-associated inflammatory pathological conditions.

Previous studies conducted at the molecular level significantly support the hypothesis that CR is capable of reducing age-associated oxidative stress and suppressing systemic, chronic inflammation [48]. CR and its anti-aging effects are majorly considered due to its significant regulatory role in oxidative stress and capability to sustain appropriate cellular redox conditions [1]. CR also has beneficial effects in the inhibition of protein synthesis and the oxidization of proteins in the liver and skeletal muscle. Furthermore, it enhances immune functions and inhibits inflammatory responses during the aging process [49]. CR exerts preventive or delaying effects on age-associated diseases, such as chronic nephropathies, cardiomyopathies, diabetes, autoimmune conditions and respiratory diseases, as well as aging [48,50]. Implementation of CR in mice suppressed the degree of neurological degeneration and β-amyloid deposition in the brain tissue and subsequently promoted the generation of neurons in in vivo animal models of Alzheimer, Parkinson, and Huntington diseases [51,52]. The results of the first randomized clinical human trial on CR highlighted a reduced probability of developing age-related diseases and improved number of biomarkers showing health longevity.

## 4. Anti-senoinflammatory Effect of CR

Many interventions and strategies for modulating chronic inflammation and anti-aging have been scientifically demonstrated. Among many well-described anti-aging strategies, CR has been identified as one of the most powerful interventions to fight the aging process and age-related pathological conditions such as diabetes, obesity, cardiovascular diseases, rheumatoid arthritis, Alzheimer’s disease and more [53]. Although the detailed molecular mechanisms and signaling pathways underlying CR still require further investigation, previous evidence for modulatory action of CR in senoinflammation suggest potential therapeutic effects of CR on aging (Table 1). For example, at a molecular level, CR exhibits powerful anti-inflammatory effects by suppressing key pro-inflammatory mediators such as NF-κB, IL-1β, IL-6, TNF, cyclooxygenase 2 (COX-2), and inducible nitric oxide synthase (iNOS) [54,55,56]. In addition, CR was shown to regulate the activity of pro-inflammatory upstream signaling pathway molecules such as MAPKs (ERK, JNK, p38), and NIK/IKKs. CR was also shown to regulate the DNA binding activity of NF-κB and AP-1 transcription factors and expression of their corresponding genes, *COX-1* and *iNOS* [55,57]. CR was also shown to reduce the plasma concentration of cytokines, TNF, ICAM-1 and to induce cortisol release, which suppresses the systemic inflammatory response [58,59]. In obese mice models, implementation of 30% CR for 2 months notably decreased the levels of adipose tissue cytokines and chemokines, including IL-6, IL-2, IL-1Rα, MCP-1, and CXCL16, which are considered as major components of SASP [60]. In hepatic tissue, even mild CR notably suppressed proinflammatory and lipogenic gene expression of molecules such as MCP-1, SREBPs, and peroxisome proliferator-activated receptor (PPAR)-γ [61]. These evidences suggest that CR successfully regulates the symptomatic prevalence of senoinflammation that expands to pathological conditions such as chronic inflammation, insulin resistance, and low energy metabolism [17,58,62,63].

Regarding anti-aging effects, CR is known to play an important role in suppressing oxidative stress and damage [64,65]. Cellular oxidative stress leads to formation of ROS, hydrogen peroxide, reactive nitrogen species, peroxynitrites, which then induce cellular inflammation, damage, and senescence. CR exerts its beneficial, maximal life-spanning effects by partially attenuating oxidative stress. For example, age-dependent functional decline of mitochondria in cardiac tissue, a major organelle of ROS production, has been well documented. Additionally, it was demonstrated that CR attenuates oxidative damage in an aged heart by lowering the levels of 8-oxodG, an oxidative damage DNA biomarker [66].

In addition to the anti-inflammatory effects described above, CR is also well known for regulating the expression of various genes involved in regulating energy metabolism. In regulating lipid metabolism, the PPARs could sense fatty acid molecules released from dietary lipids and their metabolites. PPARs are specialized receptors that recognize and bind lipid metabolites to transmit signals and can regulate lipid and carbohydrate metabolisms and inflammation. Among three subtypes of PPARs, PPARα and PPARγ have been well investigated and both have been suggested as regulators of inflammatory responses. In an aged rat model, the expression of *PPARα* and *PPARγ* genes was decreased and age-associated alterations were reversed by CR [63,67]. In a previous review, it has been noted that suppression of PPAR activity leads to upregulation of cytosolic IκB and NF-κB inhibitor, and suppression of NF-κB activation [68]. Such experimental evidence further strengthens the fact that PPARα agonists could alleviate age-related inflammation by suppressing NF-κB-mediated proinflammatory cytokine production [67,69].

CR modulates nutrient-signaling pathway molecules such as sirtuin proteins. One major molecule known to exert its effects in delaying aging and increasing longevity during CR is SIRT1 [70]. Sirtuins regulate protein expression in diverse cellular processes such as DNA repair, epigenetic modification of chromatin, ROS production, and metabolism. CR is well known to promote SIRT expression and activation in the liver, adipose tissue, brain and kidney by interacting with FOXOs, PGC1α, p53 and NF-κB to mediate anti-aging effects [71]. CR-mediated SIRT1 activity regulates pro-inflammatory NF-κB activation. For example, SIRT1 induces deacetylation and suppresses NF-κB activation [72,73].

Diverse research has provided an understanding of the association between aging and CR and the effects of CR on senoinflammatory and metabolic signaling pathways. The experimental evidence suggests that CR exerts beneficial effects on senoinflammation during aging by altering molecular pathways through regulation of expression and activities of core molecules such as NF-κB, PPARs, SIRT1, and others. Collective evidence on CR further supports the concept of senoinflammation during the aging process and confirms the positive role of CR against aging. In addition, the evidence strongly supports the notion that the anti-aging effects of CR are due to the alleviation of systematic physiological senoinflammatory response. However, further research is needed to clearly define the signaling mechanisms in detail.

## 5. Omics Big Data on Aging and CR

The immense amount of collected data in the field of biology and biomedicine research necessitates integrative data analysis to understand a complicated physiological system as a whole. Integrative dataset analysis has also provided an understanding of the underlying mechanism of aging and age-dependent changes at molecular, cellular, and physiological levels. An immense amount of data on age-related diseases enables the building of interactive networks and alterations in these networks may aid in developing aging intervention methods. Transcriptomics is the study of complete sets of RNA transcripts of a whole genome under certain conditions, which includes analysis of comparative differential gene expression in response to different conditions. As the biological aging process is complex and heterogeneous, defining specific mechanism of aging and a potential intervention method such as CR requires data integrative analysis based on age-dependent changes at the molecular level.

Among Omics Big Data analysis, gene set enrichment analysis (GSEA) allows the identification of gene types that are significantly associated with the disease. In order to analyze young and aged groups, RNA-sequencing data were collected and analyzed by the GSEA method to detect the set of differentially expressed genes or DEGs. Based on the results, it was demonstrated that proinflammatory functional genes, including cytokines, chemokines, TNF and toll-like receptors (TLRs), were notably over-represented in the aged group, whereas genes associated with metabolism, such as fatty acid metabolism and PPAR pathways, were significantly suppressed. This suggests that aging is highly associated with gene expression alterations including increased expression of inflammatory genes and decreased expression of metabolic genes. In addition, it was found that these changes were reversed by CR, a well-known aging intervention method [74].

By detecting DEGs, the Omics Big Data analysis method can identify informative epigenetic modifications along with the changes in gene expression and potential biomarkers of aging and age-related pathologic conditions. The results of a previous epigenetic study, performed using collective genomic data program called The Cancer Genome Atlas (TCGA) Program, indicated various age-related changes in the pattern of DNA hypomethylation in young and old subjects. In particular, the genes that were upregulated and hypomethylated were *AZU1*, *ELF3*, *NOX1*, *IL1B,* and *S100A12*; these genes are known to function in inflammatory responses, indicating that inflammatory genes are involved in age-related cancer onset and progression [75]. A previous briefing paper discussed the idea of big data in the field of medicine and suggested that a big network constructed by multi-omics data such as epigenomics, transcriptomics, and metabolomics can detect a significant association existing between aging and inflammation, indicating the use of a systems biology tool to identify new genes involved in inflammaging [76].

Omics Big Data is a useful tool to investigate the beneficial effects of CR. The transcriptome profile of mice liver tissue obtained after implementation of CR showed that CR improved the expression of genes associated with health, which were previously modulated by obesity. These data and other research studies collectively indicate that CR promotes beneficial outcomes leading to the expansion of life span [77]. In another study, transcriptomic data of adipose tissue indicated that CR suppresses transcription and activity of genes involved in inflammatory response, for example, the NF-κB signaling molecule gene. Additionally, in that study, diverse evidence indicated that CR has protective effects in physiological systems [78]. Furthermore, based on a global mass spectrometry-based metabolomics study, graded CR (10, 20, 30, 40% CR) modulated metabolic signaling pathways, including the carnitine synthesis and shuttle pathway and sphingosine-1-phosphate and methionine metabolism, in a graded manner. The expression of various metabolites was modulated by CR, indicating that CR could ameliorate the energy release process of hepatic fatty acids [79]. In addition, augmented gene–gene network connectivity analysis has shown that CR changed the network arrangement and biological gene centrality in a CR level-dependent manner. Therefore, the results suggested that CR-induced genes play a critical role in countering the age-associated loss of gene–gene network connectivity [80]. In order to better understand the mechanism of CR in age-associated pathological symptoms, its relation to insulin sensitivity has also been investigated. Multi-omics approaches that integrate transcriptomics, metabolomics, and microbiomics data were used to further enhance our previous knowledge that CR induced amelioration of insulin sensitivity and lifestyle, gut microbiome, and extrinsic environmental factors. Furthermore, it identified potential biomarkers that could be used for personalized weight-loss interventions [81].

Analysis of transcriptomic data from different tissues shows that aging induces the upregulation of inflammatory pathways and downregulation of metabolic pathways. In contrast, CR intervention reverses such effects, in which it downregulates the immune response and upregulates the metabolic pathways. In particular, LCK, a key signaling molecule in the development of T cells, was significantly upregulated in aged tissues and later downregulated as a consequence of CR. This identification of *LCK* gene was based on integrative analysis of cDNA microarray and interactome, which showed a high degree of centrality and between centrality analyses. These results suggest that immune and inflammatory responses are increased during aging and can be modulated by CR [82]. In support of such results, Hong et al. reported that CR successfully suppressed immune response and increased lipid metabolism, thereby delaying aging and preventing age-associated diseases [83]. Kim et al. also reported that CR implementation delayed age-associated alterations of DNA methylation, which could prevent the progression of age-related diseases [84].

Other omics studies provided evidence that CR definitively modulates aging processes and prevents or delays age-associated disease progression. Analysis of hepatic transcriptome showed that CR stimulated pathways involving IGF-1, NF-κB, mTOR, and SIRTs, which collectively contribute to reduced oxidative stress and improved metabolism, further supporting that CR promotes health and could extend lifespan by interfering with age-associated signaling pathways [85]. In addition, CR suppresses adiposity and insulin resistance, consequentially suppressing a proinflammatory status. By implementing CR, DEG analysis could help identify biomarkers that are closely related to age-associated diseases, including metabolic disease [86]. In support of the beneficial effects of CR in aging, one of the proteomics analyses demonstrated that CR improved glucose and lipid energy metabolism and suppressed oxidative stress [87]. As lipid composition was one of the critical determinants of aging, using CR as an intervention method, a research group conducted LC–MS and demonstrated that CR reprogrammed the lipidome and metabolome, which lowered the protein oxidative damage, sequentially increasing lifespan and healthspan [88].

## 6. Preventive Effects of Other Types of Dietary Restriction in Aging

CR is usually considered for reducing overall calorie intake or food intake without malnutrition. In animal models, under CR, food intake is reduced by around 10–50% compared with ad libitum-fed controls [89]. Although CR exhibits preventive effects on age-related phenotypes, to practice and sustain CR in human life is quite challenging. In efforts to improve human health during aging, there are other types of dietary restriction and pharmacological interventions available that mimic CR. Here, we introduce other types of dietary restriction and CR mimetics, recapturing the beneficial effects of CR in the present and next sections.

Reduced intake of specific nutrients, rather than reduced intake of total calories, was considered important for health benefits of a restricted diet. The reduction of either dietary protein or sugar can reduce mortality and extend life span in Drosophila, independent of the calorie intake [90]. A study by Solon-Biet et al. in mice showed a clear correlation between the ratio of protein to carbohydrate and lifespan. Mice were fed 25 diets differing systematically in protein, carbohydrate, and fat content. The energy density and median lifespan of the mice increased by up to 30% as the protein to carbohydrate ratio decreased [91]. In addition, it is suggested that reduced intake of specific essential amino acids such as methionine, tryptophan, or branched chain amino acids can delay aging or improve health [92,93].

Intermittent (ex. alternate day fasting) and periodic (fasting that lasts three days or longer, every two or more weeks) fasting have been studied as alternative dietary interventions for long-term CR [94]. The effects of fasting on lifespan extension have been reported in various species including bacteria [95], yeast [96], worm [97], and mice [98]. Intermittent fasting has a protective effect on age-dependent diseases including diabetes, cancer, heart disease, and neurodegenerative disorders in rodents [94]. There are various regimens of fasting. Recently, the fasting mimicking diet (FMD) was developed and has shown several beneficial effects in mice including extended longevity, lowered visceral fat, reduced cancer incidence and skin lesions, rejuvenated immune system, and retarded bone mineral and hippocampal neurogenesis [99].

## 7. CR Mimetics in Aging

CR mimetics are compounds that mimic the benefits of CR at the molecular, cellular, and physiological levels, leading to health-promoting effects [100]. Recently, there has been an increased interest in CR mimetics due to the benefits of using these anti-aging interventions in terms of extended health and lifespan [101,102,103]. Although the valuable effects of CR mimetics on lifespan and health have been extensively highlighted, limitations persist because it is difficult to implement such diet regimens in humans. In this section, we summarize the current knowledge of CR mimetic compounds and highlight their typical effects.

### 7.1. Resveratrol

Resveratrol (3,5,4′-Trihydroxystilbene), a natural polyphenolic, phytoalexin compound found in grapes, cranberries, and peanuts, is currently the most thoroughly studied CR mimetic. Resveratrol promotes lifespan extension across a range of evolutionarily distinct sets of species, including *Saccharomyces cerevisiae, Caenorhabditis elegans,* and *Drosophila melanogaster*, all the way to mammals such as mice [104]. Previous studies have indicated the beneficial effects of sirtuins as the best small molecule that activate sirtuins, which extended lifespan in a yeast model [104,105,106,107,108,109]. Although only the longevity extension effect of resveratrol has been reported in *C. elegans* and *D. melanogaster*, many subsequent studies reported that resveratrol intake promotes health and plays a preventive role in age-related diseases, such as cancer [110,111,112,113], atherosclerosis [114,115], arthritis [116,117], cataract [118,119,120], cardiovascular disease [121], hypertension [122,123], type 2 diabetes [124,125,126,127], osteoporosis [128,129,130], and Alzheimer disease [131,132,133]. In clinical studies, resveratrol intake improved the memory capacity of elderly individuals and reduced blood lipid levels in obese and type 2 diabetic patients [134,135]. However, additional studies are required to investigate intake duration and dose-dependent metabolic effects of resveratrol supplements required to overcome metabolic irregularity in human subjects. Resveratrol suppresses SASP through SIRT1/NF-κB signaling and delays aging [136], represses cellular senescence, and improves insulin resistance in muscle [137].

### 7.2. Metformin

Metformin, a biguanide used as a first-line drug for treating type 2 diabetes [138], was shown to extend the lifespan of *C. elegans* [139,140,141], *D. melanogaster* [142], and mice [143,144]. Moreover, it was shown to delay the onset of age-related diseases, such as cancer, metabolic syndrome [145], and cognitive disorders [146]. Its mechanism of action is associated with the activation of 5′ AMP-activated protein kinase (AMPK) [147,148], inhibition of the mammalian target of rapamycin (mTOR) [149], reduction of DNA damage [150,151], and decreased insulin levels and IGF-1 signaling [152,153,154]. The longevity effect of metformin has not yet been identified in humans, and therefore, its mechanism of action requires further investigation. Metformin regulates mitochondrial biogenesis and cellular senescence through SIRT3 [155], and decreases oxidative stress-induced senescence by activating autophagy [156].

### 7.3. Rapamycin

Rapamycin, (International Nonproprietary Name: sirolimus), is an inhibitor of mTOR, which results in an extended life span and prevents age-related diseases [157,158,159,160] by mediating SIRT1 expression [161,162]. mTOR is a serine-threonine kinase that plays a role in modulating cell survival, growth, proliferation, motility, protein synthesis and transcription [163] and inducing autophagy [164,165,166]. In addition, mTOR promotes growth and aging in *C. elegans* [167], *D. melanogaster* [168], *S. cerevisiae* [169], as well as in mice [170,171] and rat [172,173] models. Further, it modulates glucose and lipid metabolism [174,175]. Rapamycin prevents insulin resistance in humans [176], reduces insulin resistance in hyperinsulinemia rats [177,178], and normalizes glucose metabolism in diabetic mice [179,180]. Recently, Garcia et al. [181] reported that rapamycin treatment has a mechanism similar to CR in ovarian mice, which increases *FOXO3* gene expression. Thus, the use of rapamycin as a CR mimetic needs to be investigated further for understanding significant signaling pathways that can be targeted for enhancing its therapeutic potential. Rapamycin ameliorates age-related late-life cancer by inhibiting senescence-associated inflammation [182]. It is also an effective inhibitor of cellular senescence [183].

### 7.4. PPAR Agonists

In addition to the anti-inflammatory effect, PPARs have diverse biological effects including the promotion of cellular proliferation, glucose and lipid metabolism, insulin sensitivity, and tissue remodeling processes [67,184]. Because of their association with multiple metabolic processes, PPARs have been suggested to play roles in pathogenic conditions such as obesity, metabolic syndrome, diabetes, NAFLD, and atherosclerosis. Therefore, PPARs have been considered as important molecular targets for the discovery and development of new drugs to treat these age-related diseases [185,186,187,188].

Fenofibrate is a PPARα agonist used for the treatment of hyperlipidemia, hyperglycemia, and hypertriglyceridemia [189,190]. PPARα activation by fenofibrate also reduces renal oxidative stress and cellular apoptosis in aging-related renal injury through AMPK-SIRT1 and AMPK-PGC1α signaling pathways [191]. The activation of PPARα, AMPK, and SIRT1 has been shown to protect aging-related renal injury. PPARβ/δ is involved in the regulation of insulin sensitivity, adipogenesis, lipid and energy metabolism, inflammation, and atherosclerosis [192,193,194]. A specific PPARβ/δ agonist, GW501516, attenuates inflammation, insulin resistance, and dyslipidemia, and modulates angiogenesis [192,193]. Two thiazolidinediones (TZD), rosiglitazone and pioglitazone, which are also PPARγ agonists, have been shown to be effective in the treatment of type 2 diabetes [195,196]. Further, they are also associated with human life longevity and cell senescence [197]; this has also been observed in aged rats [198]. Recently, Patel et al. [199,200,201] reported that a novel dual PPARα/γ agonist, saroglitazar magnesium, was used in the treatment of dyslipidemia and metabolic disorders in in vivo and healthy Indian adult subjects. These results are further supported by the results of a preclinical study conducted by Kaul et al. [201]. Notably, Xu et al. [202] reported that chiglitazar acts as a PPAR-α/β/γ pan agonist and evaluated its use in diabetic therapeutics in healthy Chinese volunteers.

Recent data support PPAR agonists as potential candidates for anti-senoinflammation therapy. Our group synthesized MHY908, new a PPARα/γ dual agonist, and showed that it has a significant inhibitory effect on age-related inflammation and insulin resistance [203]. It is reported that PPAR activation might have an effect on the prevention of cell senescence and that PPARα silencing induces cancer cell senescence. Rosiglitazone significantly suppressed olaparib (a PARP inhibitor)-induced cellular senescence and SASP in ovarian cancer [204].

Collectively, identifying the role of PPAR agonists in various metabolic or non-metabolic organs and pathological conditions will contribute to the development of new therapeutic options and promising anti-senoinflammatory chemicals for the treatment of many age-related metabolic disorders.

### 7.5. Ketone Bodies

Ketone bodies such as β-hydroxybutyrate (HB), acetoacetate, and acetone are water-soluble molecules that are generated from fatty acids in the mitochondrial matrix of the liver. They serve as moving energy sources for physiological systems during periods of fasting [205]. The process of ketogenesis starts within 24 h of fasting through gluconeogenesis [206]. In humans, physiological serum levels of HB are normally maintained at a low micromolar concentration, which increases to a few hundred micromoles after 12 to 16 h of the fasting period and eventually reaches 1 to 2 mM after 2 days of fasting [207]. Insulin inhibits lipolysis of adipose tissue and restricts ketogenesis, while glucagon promotes ketogenic flow by exerting its direct effect on the hepatic tissue [208]. In a study, CR or fasting interventions elevated the circulating concentration of ketone bodies, HB, compared to that in a normal feeding group [209,210]. Furthermore, it has also been reported that the implementation of a ketogenic diet exerts therapeutic effects on various age-related diseases related to insulin resistance, as well as diseases resulting from free radical damage and hypoxia [211].

HB also acts as a signaling molecule and activates cellular signaling pathways. For example, HB plays a role in endogenously inhibiting histone deacetylases (HDACs) [209]. Suppression of HDAC activity exerts beneficial metabolic and cytoprotective effects similar to those seen in HB investigations [212]. However, SIRT3 regulates diverse pathways involved in fasting metabolism, and mice without *SIRT3* genes have decreased HB concentration during fasting [188]. Ketogenic diets are also related to low levels of insulin [213,214], suppressed IGF signaling [215], induction of FOXO3 [209], and activation of AMPK [215,216] and antioxidant genes [209]. Ketone bodies exert neuroprotective and lifespan extension effects similar to CR in *C. elegans* [217]. HB upregulates transcription of antioxidant genes, including manganese superoxide dismutase (*MnSOD*) and *FOXO3*, both of which exert antioxidant effects [209]. It is thought that HB exerts its effect through signaling mechanisms comparable to that of CR by inducing co-activation of FOXO1/PGC-1α through deactivation of the PI3K/Akt pathway [218].

HB is an effector that transduces signals via G-protein coupled receptors. It represses the actions of the sympathetic nervous system and decreases energy expenditure and heart rate by blocking fatty acid signaling pathways through the G protein-coupled receptor 41 [219]. One of the most well-studied signaling effects of HB signals is via GPR109A, a member of the hydrocarboxylic acid GPCR subfamily that is expressed in adipose tissues (white and brown) [220] and immune cells [221]. Although the GPR109A receptor has protective effects, associations have been found between ketogenic dietary intervention use in stroke patients and neurodegenerative diseases [222,223]. In a TNFα or LPS-induced inflammatory setting, HB exerts anti-inflammatory effects by suppressing the release of pro-inflammatory proteins (iNOS and COX-2) and cytokines (TNF, IL-1β, IL-6 and CCL2/MCP-1), which seems to occur partially via inhibition of NF-κB translocation to the nucleus for pro-inflammatory gene activation [224,225]. However, in neurodegenerative inflammatory conditions, the effects of GPR109A-mediated HB do not appear to involve inflammatory mediator signaling via the MAPK pathway [224]. In addition to their role in providing energy fuels for various key organs and tissues, including the brain, heart or skeletal muscle, ketone bodies play critical roles as signaling mediators and modulators of inflammation and oxidation [226].

## 8. Conclusions

Based on the available molecular and biochemical evidence, we proposed the concept of senoinflammation in our previous review [17,227]. The concept proposes a broader perspective on age-related inflammatory response and creates a complex network among many inflammatory mediators that can lead to systemic chronic inflammation. Oxidative stress leads to improper gene regulation and genomic DNA damage during aging. Such improper gene regulation in aged senescent cells allows them to fall into a proinflammatory state, consequently changing systemic chemokine or cytokine activities. The proinflammatory SASP environment further exerts stress on the intracellular organelles, tissues, and systems, which affects the development and occurrence of metabolic disorders. It appears that a repetitive vicious cycle occurs between SASP and metabolic dysregulation as proposed in the concept of senoinflammation, and this interactive network forms the basis of the aging process and age-related diseases. However, the secretion of proinflammatory mediators, collectively termed as SASP, in response to internal and external stress leads to the chronic inflammatory condition termed as senoinflammation. Based on CR experiments and observations, cytokine, chemokine, and metabolic pathways are significantly regulated by CR and CR mimetics in the aging process. It is expected that a better understanding of senoinflammation modulatory mechanisms will provide a basis for the discovery of molecular targets that can therapeutically modulate age-related chronic inflammatory conditions and enable the development of potentially effective interventions to delay aging and prevent the occurrence of aging-associated diseases.

## Figures and Tables

**Table 1:** Changes in parameters in senoinflammation.

	SASP Factors	Old	CR	Species	References
Cytokines	IL-1β	↑	├	Human, Mouse, Rat	[82,228,229,230]
	IL-6	↑	├	Human *, Mouse, Monkey	[82,231,232,233]
	IL-7	↑		Human, Rat	[74,82], TCGA database
	IL-13	-		Human	TCGA database
	IL-11	↑		Rat	[74,82]
	IL-6R	↑		Rat	[74,82]
	IL-2RA	↑		Rat	[74,82]
	TNF-α	↑	├	*C. elegans*, Mouse, Rat	[74,82,231,234]
	TNF-β	↑		Human, Rat	[74,82], TCGA database
Cheomokines	IL-8	↑	├	Monkey	[235]
	MCP-1 (CCL2)	↑	├	Mouse, Rat	[74,82,236]
	MCP-2	-	├	Mouse	[237]
	MIP-1α (CCL3)	-	├	Mouse	[236,238]
	MIP-3α	↑		Rat	[74,82]
MMPs, GFs, etc.	MMP1	↑		Human, Mouse	[239,240]
	MMP2	↑	├	Mouse	[241]
	MMP3	↑	├	Mouse, Rat	[60,74,82,242]
	MMP9	↑	├	Mouse, Rat	[243,244]
	MMP12	↑		Rat	[74,82]
	MMP13	↑	├	Rat	[245]
	MMP14	↑		Human	TCGA database
	HGF	↑		Human, Rat	[74,82], TCGA database
	EGFR	↑		Human, Rat	[74,82], TCGA database
	FAS	↑	├	Human, Mouse, Rat	[74,82,246,247,248]
	IGFBP2	↑		Human	TCGA data base
Metabolism	Insulin resistance	↑	├	Human, Mouse, Rat	[181,249,250,251]
	ER stress	↑	├	Human, Mouse, Rat	[252,253,254]
	Autophagy	↑	├	Human, Mouse, Rat	[255,256,257,258]
	Lipid accumulation	↑	├	Human, Mouse, Rat	[259,260,261,262]

* A calorie restriction (CR) diet supplemented with fish oil. SASP, senescence-associated secretory phenotype; IL-1β, Interleukin 1 beta; IL-6R, Interleukin 6 receptor; TNF-α, Tumor necrosis factor-alpha; MCP-1, Monocyte chemoattractant protein-1; MIP-1α, Macrophage inflammatory protein-1alpha; MMP, Matrix metallopeptidases; GF, Growth factor; HGF, Hepatocyte Growth Factor; EGFR, Epidermal growth factor receptor; FAS, Apoptosis Antigen 1; IGFBP2, Insulin Like Growth Factor Binding Protein 2; TCGA, The Cancer Genome Atlas.
